# Distinction between vaginal and cervical microbiota in high-risk human papilloma virus-infected women in China

**DOI:** 10.1186/s12866-021-02152-y

**Published:** 2021-03-25

**Authors:** Zhan Zhang, Ting Li, Dai Zhang, Xiaonan Zong, Huihui Bai, Hui Bi, Zhaohui Liu

**Affiliations:** 1 The Gynecology Department of Beijing Obstetrics and Gynecology Hospital, Capital Medical University, No. 251 of Yaojiayuan Road, Chaoyang District, Beijing, China; 2 The Gynecology Department of Peking University First Hospital, No. 1 of Xi’anmen Street, Xicheng District, Beijing, China; 3 The Microecological Laboratory of Beijing Obstetrics and Gynecology Hospital, Capital Medical University, No. 251 of Yaojiayuan Road, Chaoyang District, Beijing, China

**Keywords:** High-risk human papilloma virus, Vaginal microbiota, Cervical microbiota, Difference

## Abstract

**Background:**

High-risk human papilloma virus (hrHPV) is the main causal factor of cervical precancer and cancer when persistent infection is left untreated. Previous studies have confirmed the vaginal microbiota is associated with HPV infection and the development of cervical lesions. The microbiota at different parts of the female genital tract is closely related but different from each other. To analyze the distinction between the vaginal and cervical microbiota of hrHPV(+) women in China, one hundred subjects were recruited, including 10 patients with HPV16/18(+) and cervical carcinoma, 38 patients with HPV16/18(+) but no cervical carcinoma, 32 patients with other hrHPV(+) and 20 healthy controls with HPV(−). Vaginal and cervical microbiota were separately tested through next-generation sequencing (NGS) targeting the variable region (V3-V4) of the bacterial ribosome 16S rRNA gene.

**Results:**

HrHPV(+) subjects had higher percentages of vaginal douching history (*P* = 0.001), showed more frequent usage of sanitary pads (*P* = 0.007), had more sex partners (*P* = 0.047), were more sexually active (*P* = 0.025) and more diversed in ways of contraception (*P* = 0.001). The alpha diversity of the cervical microbiota was higher than that of the vagina. The cervical microbiota consisted of a lower percentage of *Firmicutes* and a higher percentage of *Proteobacteria* than the vagina at the phylum level. *Sphingomonas*, belonging to α-*Proteobacteria,* was almost below the detection limit in the vagina but accounted for five to 10 % of the bacteria in the hrHPV(−) cervix (*P*<0.001) and was inversely associated with hrHPV infection (*P*<0.05). *Pseudomonas*, belonging to γ-*Proteobacteria,* could hardly be seen in the normal vagina and shared a small percentage in the normal cervix but was significantly higher in the HPV16/18(+) (*P*<0.001) and cancerous cervix (*P*<0.05). No significant difference was shown in the percentage of BV associated anaerobes, like *Gardnerella*, *Prevotella*, *Atopobium* and *Sneathia*, between the cevix and vigina.

**Conclusions:**

The proportion of *Proteobacteria* was significantly higher in the cervical microbiota than that of vagina. The hrHPV infection and cervical cancer was positively associated with *Pseudomonas* and negatively associated with *Sphingomonas*. It is of great improtance to deeply explore the cervical microbiota and its function in cervical cacinogenesis.

## Background

Human papilloma virus (HPV) is a double-stranded DNA virus that only infects the human body, and two types have been identified, namely, the skin type and mucosal type, comprising over 100 subtypes [1]. More than 40 mucosal types of HPV can infringe on the human reproductive system, and 15 high-risk HPV (hrHPV) types have been demonstrated to be related to cervical lesions: HPVs 16, 18, 31, 33, 35, 39, 45, 51, 52, 53, 56, 58, 59, 66 and 68 [1]. Approximately 50 and 20% of cervical cancers are induced by HPV 16 and 18, respectively, and these two subtypes are considered to be extremely high-risk types [2]. Persistent HPV infection plays a pivotal role in cervical cancer development. The progression from hrHPV infection to squamous intraepithelial lesion (SIL) and cancer can generally last years or even decades [3], and within the time window, preventative or therapeutic intervention can be executed.

As the world’s largest developing country, approximately 300 million women in China need cervical cancer screening every year. The primary prevention strategy represented by the HPV vaccine in China is still in its infancy [4]. China has not incorporated the HPV vaccine in the National Immunization Program. After the United States licensure in 2006, the first HPV vaccine was approved in China until 2016. Currently, information about vaccines and their acceptance among Chinese women is scarce [4]. The current situation in the primary prevention of hrHPV is still not optimistic. Therefore, we should strengthen the efforts of secondary and tertiary prevention and look for deeper factors of hrHPV infection and cervical lesions, which will help to provide a theoretical basis for innovation in the control of hrHPV and cervical lesions.

In addition to HPV infection, several other related factors were also involved in cancerous progression. Studies [5, 6] have paid attention to the following factors and confirmed their association with cervical lesions: socioeconomic factors, hygienic habits, sexual and parity, etc. With the development of microbiological detection technology, especially next-generation sequencing (NGS), there have been increasing concerns in recent years that the genital microbial environment may be associated with HPV infection and cervical lesions [7].

It has been recently proposed that abnormal vaginal microbiota plays a significant role in the development of cervical neoplasms. In the female lower reproductive tract, health is more commonly associated with low microbial diversity and dominance by only one or a few species of *Lactobacillus* [8]. Lee [9] analyzed the relationship between HPV infection and vaginal flora for the first time and discovered a higher diversity of vaginal microbiota in HPV-infected women. Soon after, Brotman [10] found that the type of vaginal microbiota might be associated with HPV clearance or persistence. Thus, many experts have begun to pay attention to microdysbiosis of the female lower genital tract and infer that vaginal microbiota disturbance might directly relate to HPV acquisition and even to cervical cancer. A very recent study [11] revealed that microbiota at different parts of the female genital tract might be closely related but distinct from each other, changing from the vagina to the cervix, endometrium, fallopian tubes and peritoneal fluid. Most of the studies to date preferred to refer the sample as “cervicovaginal” instead of discussing “cervical” and “vaginal” samples. No such research has been done on the distinction between the cervical and vaginal microbiota of hrHPV-infected women in the Chinese population, which has led us to design this project to explore the continuum and distinction between the cervical and vaginal microbiota in hrHPV-infected women in China. This research would be meaningful for further research to explore the role of microbiota in cervical carcinogenesis [12].

## Results

### Demographics

To characterize the cervical and vaginal microbiota in hrHPV(+) Chinese women, we obtained vaginal and cervical samples from 100 subjects and divided them into four groups, namely, the normal/control group (Group N, *n* = 20), the other hrHPV group (Group O, *n* = 32), HPV 16/18 group (Group H, *n* = 38) and cervical cancer group (Group C, *n* = 10). HPV 16/18 and other high-risk subtypes were specifically separated since HPV 16/18 are extremely high-risk subtypes and cause nearly 70 % cervical carcinoma [2]. Subjects of each group were age matched (Table 1, *P* = 0.289). All enrolled subjects underwent regular TCT and HPV tests (Table 1), some of whom might be infected with multiple subtypes. Prevalence of HPV 16 and 18 were 81.6 and 47.4%, respectively, in which some were coinfected with both. The other frequent HPV subtypes were HPV 52 (37.5%), 58 (15.6%), 33 (12.5%) and 53 (12.5%). Cancerous subjects were diagnosed with cervical squamous carcinoma staged from Federation of International Gynecology and Obstetrics (FIGO) Ia1 to IIa2 (Table 1). Subjects in groups H and C tended to have more frequent usage of sanitary pads (*P* = 0.007), more sex partners (*P* = 0.047), more frequent intercourse (*P* = 0.025) and more accustomed to vaginal douching (*P* = 0.001). People infected with hrHPV had a higher proportion of vaginitis history (*P* = 0.002). Condom use was significantly lower and contraceptive methods were more varied in hrHPV(+) individuals (*P* = 0.001).

**Table 1:** Demographics of participants

	Normal (*n* = 20)	Other hrHPV(+) (*n* = 32)	HPV16/18(+) (*n* = 38)	Cervical cancer (*n* = 10)	P*
Mean age (years)	38.35 ± 3.72	36.75 ± 6.15	35.73 ± 6.89	38.80 ± 3.08	0.289
TCT					
Normal	20	9 (28.1%)	22 (57.9%)	-^a^	
Ascus	–	11 (34.3%)	3 (7.9%)	–	
LSIL	–	8 (25.0%)	4 (10.5%)	–	
HSIL	–	2 (6.3%)	7 (18.4%)	–	
ASC-H	–	2 (6.3%)	2 (5.3%)	–	
HPV subtypes^b^					
16	–	–	31 (81.6%)	8 (80.0%)	
18	–	–	8 (47.4%)	3 (30.0%)	
16and18	–	–	1 (2.6%)	1 (10.0%)	
31	–	1 (3.1%)	1 (2.6%)	0	
33	–	4 (12.5%)	2 (5.3%)	0	
35	–	1 (3.1%)	0	1 (10.0%)	
51	–	2 (6.3%)	3 (7.9%)	0	
52	–	12 (37.5%)	3 (7.9%)	0	
53	–	4 (12.5%)	2 (5.3%)	0	
56	–	3 (9.4%)	2 (5.3%)	0	
58	–	5 (15.6%)	2 (5.3%)	0	
59	–	3 (9.4%)	0	0	
66	–	2 (6.3%)	0	0	
68	–	3 (9.4%)	0	0	
Vaginitis or not					0.002
Normal	16 (80.0%)	27 (84.4%)	25 (65.8%)	3 (30.0%)	
Bacterial vaginosis	2 (10.0%)	5 (15.6%)	13 (34.2%)	5 (50.0%)	
Abnormal flora	1 (5.0%)	0	0	1 (10.0%)	
Flora suppression	1 (5.0%)	0	0	1 (10.0%)	
Leukocytes at cervix					0.001
0–10	14 (70.0%)	12 (37.5%)	12 (31.6%)	1 (10.0%)	
>10	6 (30.0%)	20 (62.5%)	26 (68.4%)	9 (90.0%)	
Cervical biopsy					0.129
Normal	20	0	0	–	
Cervicitis	–	14 (43.8%)	11 (28.9%)	–	
LSIL	–	11 (34.4%)	10 (26.3%)	–	
HSIL	–	7 (21.8%)	17 (44.8%)	–	
Cancer	–	0	10	10	
Educational level					0.478
<Bachelor	9 (45.0%)	12 (37.5%)	17 (44.7%)	5 (50.0%)	
Bachelor	10 (50.0%)	19 (59.4%)	15 (39.5%)	5 (50.0%)	
≥ Master	1 (5.0%)	1 (3.1%)	6 (15.8%)	0	
Monthly income (¥)					0.295
<5000	8 (40.0%)	9 (28.1%)	19 (50.0%)	6 (60.0%)	
5000–10,000	10 (50.0%)	17 (53.1%)	12 (31.6%)	4 (40.0%)	
>10,000	2 (10.0%)	6 (18.8%)	7 (18.4%)	0	
Occupation					0.838
Medical service	2 (10.0%)	2 (6.3%)	0	0	
Economics	2 (10.0%)	5 (15.6%)	5 (13.2%)	0	
Education	1 (5.0%)	2 (6.3%)	4 (10.5%)	1 (10.0%)	
Art	1 (5.0%)	1 (3.1%)	1 (2.6%)	0	
Worker/Farmer	14 (70.0%)	22 (68.7%)	28 (73.7%)	9 (90.0%)	
Frequency of cleaning vulva					0.306
<1 time per week	0	4 (12.5%)	3 (7.9%)	0	
2–3 times per week	6 (30.0%)	5 (15.6%)	10 (26.3%)	5 (50.0%)	
Everyday	14 (70.0%)	23 (71.9%)	25 (65.8%)	5 (50.0%)	
Way of cleaning vulva					0.195
Clean water	19 (95.0%)	23 (71.9%)	31 (81.6%)	9 (90.0%)	
Lotion	1 (5.0%)	9 (28.1%)	7 (18.4%)	1 (10.0%)	
History of vaginal douching					0.001
Yes	0	11 (34.4%)	19 (50.0%)	4 (40.0%)	
No	20 (100.0%)	21 (65.6%)	19 (50.0%)	6 (60.0%)	
Days of using sanitary pads					0.007
≥ 10 days per month	2 (10.0%)	10 (31.3%)	9 (23.7%)	7 (70.0%)	
<10 days per month	18 (90.0%)	22 (68.7%)	29 (76.3%)	3 (30.0%)	
Smoking or not					0.710
Yes	0	2 (6.3%)	1 (2.6%)	0	
No	20 (100.0%)	30 (93.7%)	37 (97.4%)	10 (100.0%)	
Age of first sex					0.152
<20 years old	1 (5.0%)	9 (28.1%)	5 (13.2%)	1 (10.0%)	
≥ 20 years old	19 (95.0%)	23 (71.9%)	32 (86.8%)	9 (90.0%)	
Number of sex partner					0.047
1	18 (90.0%)	15 (46.8%)	21 (55.3%)	7 (70.0%)	
2	2 (10.0%)	11 (34.4%)	13 (34.2%)	3 (30.0%)	
≥ 3	0	6 (18.8%)	4 (10.5%)	0	
Frequency of sex					0.025
< 1 time per week	16 (80.0%)	20 (62.5%)	20 (52.7%)	2 (20.0%)	
2–3 times per week	4 (20.0%)	9 (28.1%)	14 (36.8%)	8 (80.0%)	
≥ 3 times per week	0	3 (9.4%)	4 (10.5%)	0	
Methods of contraception^c^					0.001
None	2 (10.0%)	2 (6.3%)	5 (13.2%)	2 (20.0%)	
Condom	15 (75.0%)	12 (37.5%)	13 (34.2%)	1 (10.0%)	
Oral contraceptive	0	1 (3.1%)	2 (5.3%)	0	
Intrauterine device	3 (15.0%)	3 (9.4%)	6 (15.8%)	5 (50.0%)	
Others	0	14 (43.7%)	12 (31.6%)	2 (20.0%)	
Parity					0.955
≤ 1 time	18 (90.0%)	30 (93.8%)	35 (92.1%)	9 (90.0%)	
≥ 2 times	2 (10.0%)	2 (6.2%)	3 (7.9%)	1 (10.0%)	
Number of abortion					0.222
≤ 1 time	12 (60.0%)	27 (84.4%)	30 (78.9%)	7 (70.0%)	
≥ 2 times	8 (40.0%)	5 (15.6%)	8 (21.1%)	3 (30.0%)	
History of vaginitis					0.002
Yes	2 (10.0%)	14 (43.7%)	21 (55.3%)	7 (70.0%)	
No	18 (90.0%)	18 (56.3%)	17 (44.7)	3 (30.0%)	

*Abbreviations: hrHPV* high-risk human papilloma virus, *TCT* thin-prep cytology test, *Ascus* atypical squamous cell of undetermined significance, *LSIL* low-grade squamous intraepithelial lesion, *HSIL* high-grade squamous intraepithelial lesion, *ASC-H* atypical squamous cell- cannot exclude HSIL

*: *P* values were calculated by ANOVA, Chi-square test or Fisher exact probability test

^a^: “-” means no sense; ^b^: HPV subtypes might overlap in each participant; ^c^: Methods of contraception means the most common way of birth control

### The diversity of the cervical microbiota was different from that of the vagina

**Fig. 1:**
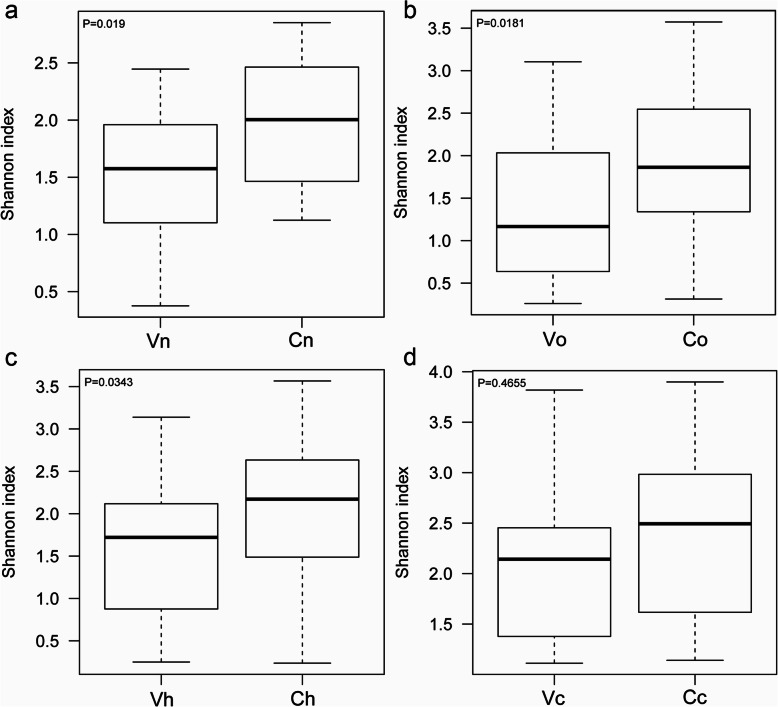
Alpha diversity of vaginal and cervical microbiota. The greater the Shannon value, the higher the diversity of the community. Vn, Vo, Vh and Vc represent the vaginal microbiota of groups N (the normal control group), O (the other hrHPV group), H (the HPV16/18 group) and C (the cancer group), respectively. Cn, Co, Ch and Cc represent the cervical microbiota of groups N, O, H and C, respectively. (**a**, group N, *n* = 20, *P* = 0.019. **b**, group O, *n* = 32, *P* = 0.0181. **c**, group H, *n* = 38, *P* = 0.0343. **d**, group C, *n* = 10, *P* = 0.4655)

**Fig. 2:**
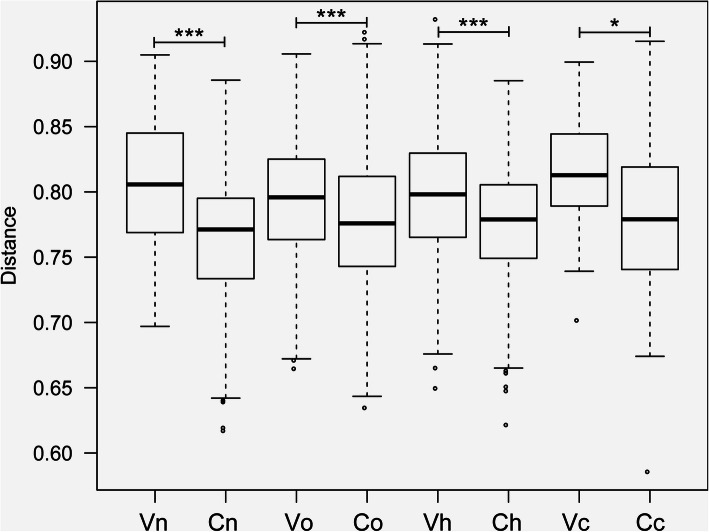
Beta diversity of vaginal and cervical microbiota. UniFrac distance reflects evolutionary distances of strains, ranging from 0 to 1. Vn, Vo, Vh and Vc represent the vaginal microbiota of groups N (the normal control group), O (the other hrHPV group), H (the HPV16/18 group) and C (the cancer group), respectively. Cn, Co, Ch and Cc represent the cervical microbiota of groups N, O, H and C, respectively. (*, *P* <0.05; **, *P* <0.01; ***, *P* <0.001)

Herein, the cervical microbiota was separately discussed from the vaginal microbiota. For convenience, the vaginal microbiota of the four groups was abbreviated as Vn, Vo, Vh and Vc, and the cervical microbiota was correspondingly abbreviated as Cn, Co, Ch and Cc. Subscripts such as “n, o, h and c” represent the normal group, the other hrHPV(+) group, the hrHPV16/18 group and the cancer group, respectively. We used the Shannon index to represent the alpha diversity of species. The higher the Shannon index was, the more diverse the microbiota in the sample. We observed the Shannon index of Cn was significantly higher than that of Vn, demonstrating a higher microbial diversity of the healthy cervix than the vagina (Fig. 1a, *P* =0.019). The same trends were observed in Co vs Vo (Fig. 1b, *P* =0.018) and Ch vs Vh (Fig. 1c, *P* =0.034). No significant difference was found between Cc and Vc (Fig. 1d, *P* =0.466). To clarify whether the cervical microbiota is different from the vaginal microbiota, beta diversity analysis was also performed. In this part, the UniFrac distance was calculated to estimate the evolutionary differences of species between different groups. The boxplot showed a significant difference between the cervical and vaginal microbiota (Fig. 2). Vn vs Cn, *P* <0.001; Vo vs Co, *P*<0.001; Vh vs Ch, *P*<0.001; Vc vs Cc, *P*<0.05), which revealed that there existed different communities in the vagina and cervix from an evolutionary perspective.

### *Proteobacteria* was much more prevalent in the cervix than in the vagina

**Fig. 3:**
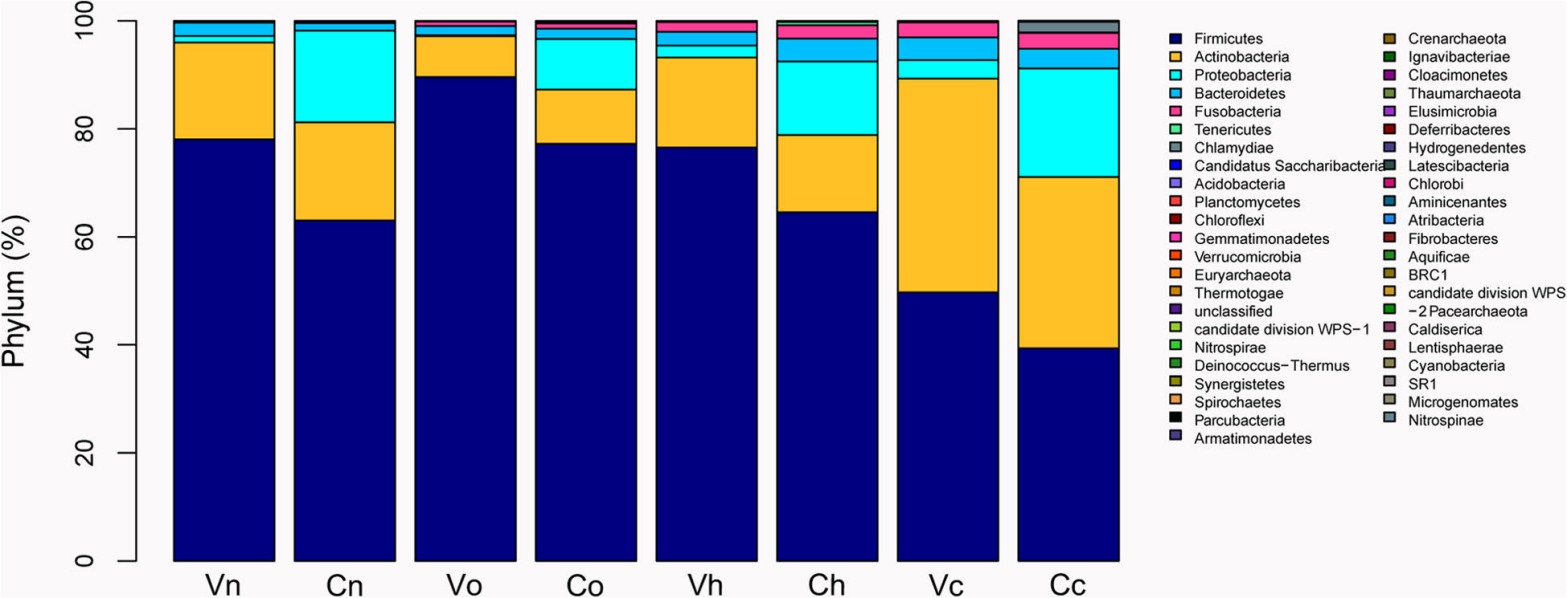
Vaginal and cervical microbiota distribution at the phylum level. The abscissa denotes groups, and the ordinate denotes the percentage of microbes at the phylum level. Vn, Vo, Vh and Vc represent the vaginal microbiota of groups N (the normal control group), O (the other hrHPV group), H (the HPV16/18 group) and C (the cancer group), respectively. Cn, Co, Ch and Cc represent the cervical microbiota of groups N, O, H and C, respectively

**Fig. 4:**
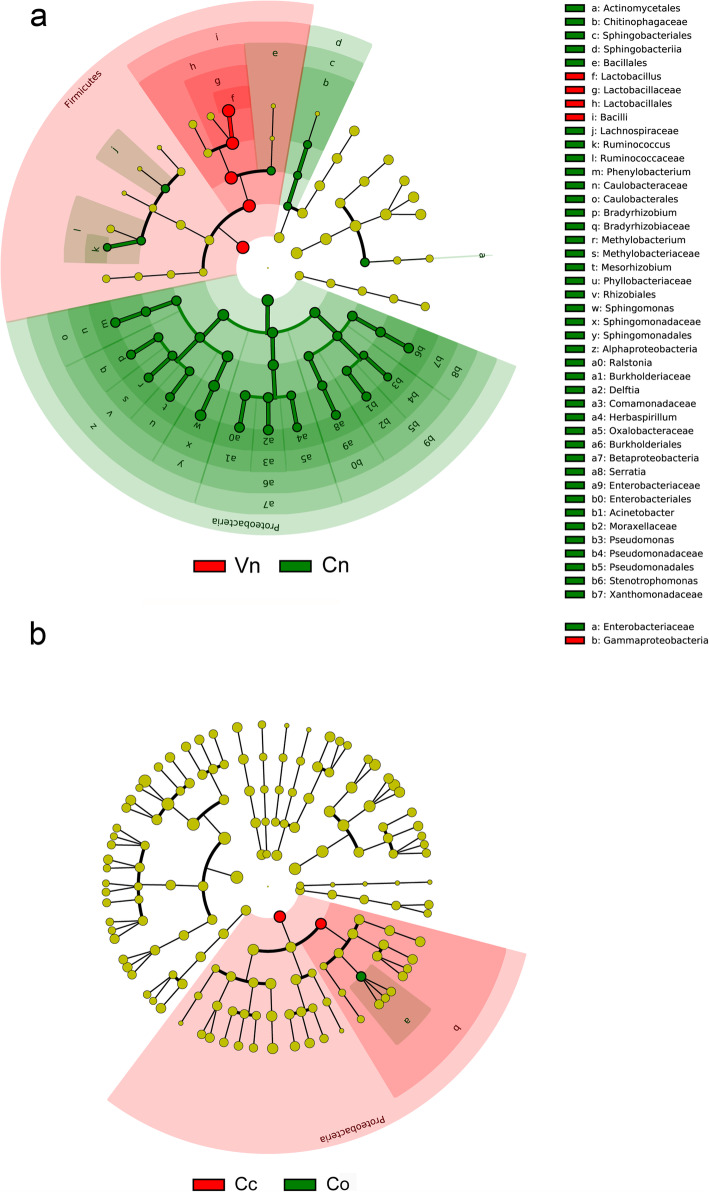
LEfSe linear discriminant analysis of vaginal and cervical microbiota. In the LEfSe cladogram, different colors in the branches represent microbes associated with the relevant group. Vn, Vo, Vh and Vc represent the vaginal microbiota of groups N (the normal control group), O (the other hrHPV group), H (the HPV16/18 group) and C (the cancer group), respectively. Cn, Co, Ch and Cc represent the cervical microbiota of groups N, O, H and C, respectively. (**a**, Vn vs Cn. **b**, Comparison of Vn, Vo, Vh, Vc, Cn, Co, Ch and Cc)

The vaginal and cervical microbiota mainly consisted of six major phyla, namely, *Firmicutes*, *Actinobacteria*, *Bacteroidetes*, *Fusobacteria*, *Proteobacteria* and *Tenericutes* (Fig. 3). Linear discriminant analysis effect size (LEfSe) analysis showed a lower percentage of *Firmicutes and* a higher percentage of *Proteobacteria* in the normal cervix than in the vagina in HPV(−) subjects (Fig. 4a), which indicated that *Proteobacteria* was a special phylum in the normal cervix. To explore the particularity of *Proteobacteria* in the hrHPV(+) cervix, we further compared the vaginal and cervical microbiota of the four groups and observed that γ-*Proteobacteria* was more abundant in the cancerous cervix (Fig. 4b).

**Fig. 5:**
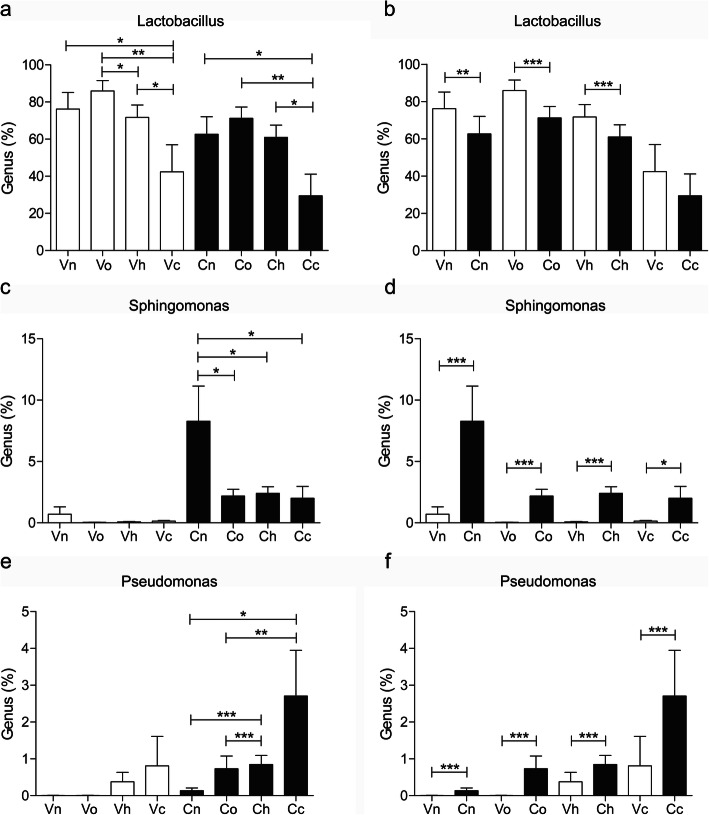
Distribution of *Lactobacillus*, *Sphingomonas* and *Pseudomonas* in the vagina and cervix of the subjects. Vn, Vo, Vh and Vc represent the vaginal microbiota of groups N (the normal control group), O (the other hrHPV group), H (the HPV16/18 group) and C (the cancer group), respectively. Cn, Co, Ch and Cc represent the cervical microbiota of groups N, O, H and C, respectively. (*, *P* <0.05; **, *P* <0.01; ***, *P* <0.001)

To identify the target genus residing in the cervix associated with hrHPV infection and cervical cancer, we systematically compared some representative bacteria in the vagina and cervix of hrHPV(+) subjects, such as *Lactobacillus*, *Sphingomonas*, *Pseudomonas* and bacterial vaginosis (BV) -related anaerobes such as *Gardnerella*, *Prevotella*, *Atopobium* and *Sneathia*. We observed that depleted *Lactobacillus* was associated with cervical cancer (Fig. 5a, Vn vs Vc, *P*<0.05; Cn vs Cc, *P*<0.05; Vo vs Vc, *P*<0.01; Co vs Cc, *P*<0.01; Vh vs Vc, *P*<0.05; Ch vs Cc, *P*<0.05). A lower level of *Lactobacillus* was seen in the cervix than in the vagina in both hrHPV(+) and hrHPV(−) subjects, which was not significant in the cancerous cervix. (Fig. 5b, Vn vs Cn, *P*<0.01; Vo vs Co, *P*<0.001; Vh vs Ch, *P*<0.001).

*Sphingomonas* and *Pseudomonas* were selected as representative genera in *Proteobacteria* because we found their association with hrHPV infection and cervical cancer. *Sphingomonas*, which belongs to α-*Proteobacteria*, was almost below the detection limit in the vagina (Fig. 5c) but accounted for five to 10 % of the bacteria in the hrHPV(−) cervix (Fig. 5d, Vn vs Cn, *P*<0.001) and was inversely associated with hrHPV infection (Fig. 5c, Cn vs Co/Ch/Cc, *P*<0.05). *Pseudomonas* could hardly be seen in the normal vagina and shared a small percentage in the HPV16/18(+) and cancerous vagina (Fig. 5e). However, *Pseudomonas* was relatively high in the HPV16/18(+) and cancerous cervix (Fig. 5e, Cn vs Ch, *P*<0.001; Co vs Ch, P<0.001; Cn vs Cc, *P*<0.05; Co vs Cc, *P*<0.01. Figure 5f, Vh vs Ch, *P*<0.001; Vc vs Cc, *P*<0.001).

**Fig. 6:**
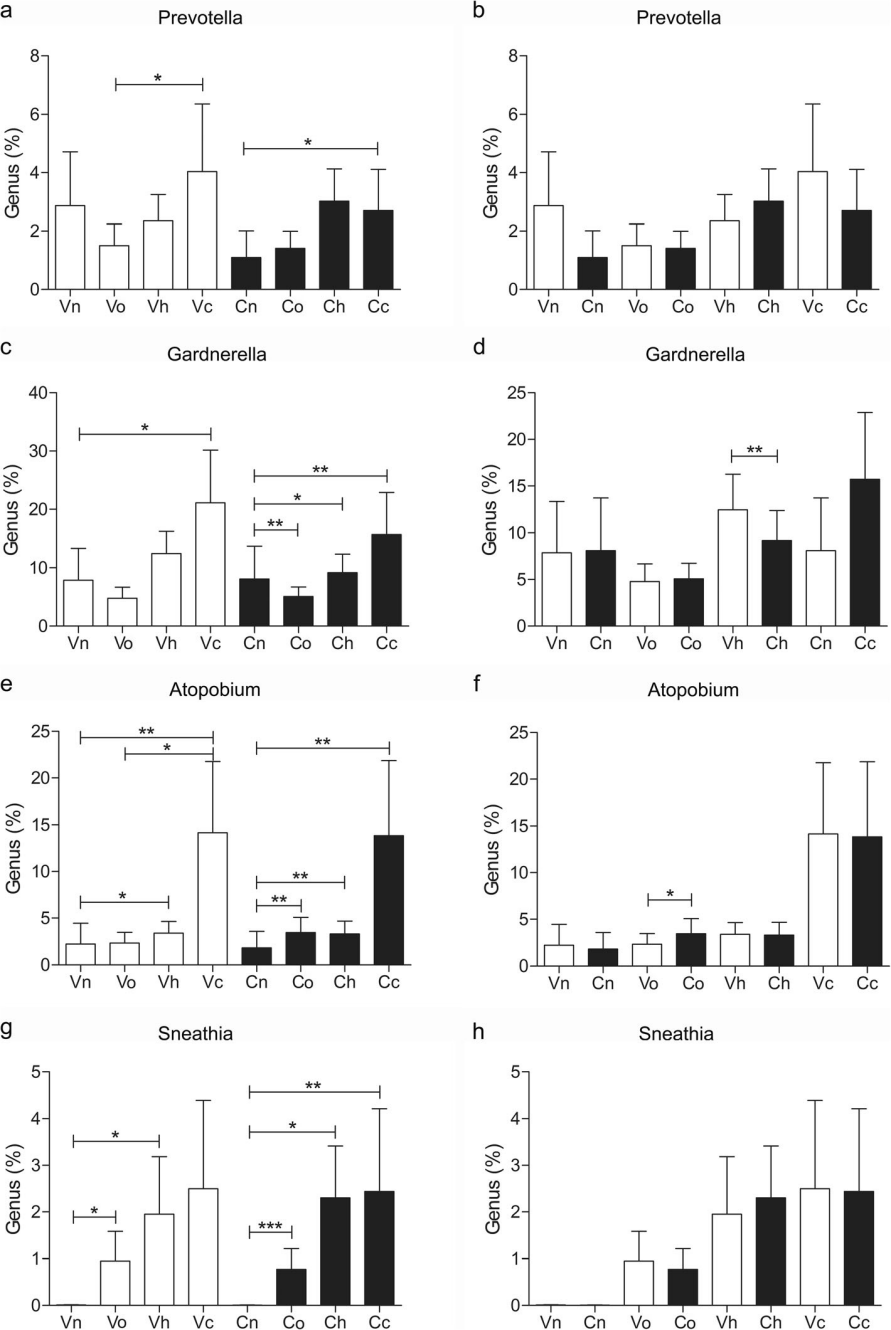
Distribution of *Prevotella*, *Gardnerella*, *Atopobium* and *Sneathia* in the vagina and cervix of the subjects. Vn, Vo, Vh and Vc represent the vaginal microbiota of groups N (the normal control group), O (the other hrHPV group), H (the HPV16/18 group) and C (the cancer group), respectively. Cn, Co, Ch and Cc represent the cervical microbiota of groups N, O, H and C, respectively. (*, *P* <0.05; **, *P* <0.01; ***, *P* <0.001)

BV-related anaerobes showed similar changes in both the vaginal and cervical microbiota of hrHPV(+) subjects and did not exhibit “cervical specificity” (Fig. 6b, d, f, h). *Prevotella* was higher mainly in the cancerous vagina and cervix (Fig. 6a, Vo vs Vc, *P*<0.05; Cn vs Cc, *P*<0.05). *Gardnerella* shared a higher percentage in the HPV16/18(+) cervix and cancerous vagina/cervix (Fig. 6c, Vn vs Vc, *P*<0.05; Cn vs Cc, *P*<0.01; Cn vs Ch, *P*<0.05). *Atopobium* was higher in HPV16/18(+) and cancerous vagina/cervix (Fig. 6e, Vn vs Vh, *P*<0.05; Vn vs Vc, *P*<0.01; Cn vs Ch, *P*<0.01; Cn vs Cc, *P*<0.01) and more prevalent in other hrHPV-infected cervixes (Fig. 6e, Cn vs Co, *P*<0.01). *Sneathia* was significantly higher in all hrHPV-infected vagina/cervix regardless of subtypes (Fig. 6g, Vn vs Vo, *P*<0.05; Vn vs Vh, *P*<0.05; Cn vs Co, *P*<0.001; Cn vs Ch, P<0.05; Cn vs Cc, P<0.01).

### The function of the cervical microbiota was more active than that of the vagina

**Fig. 7:**
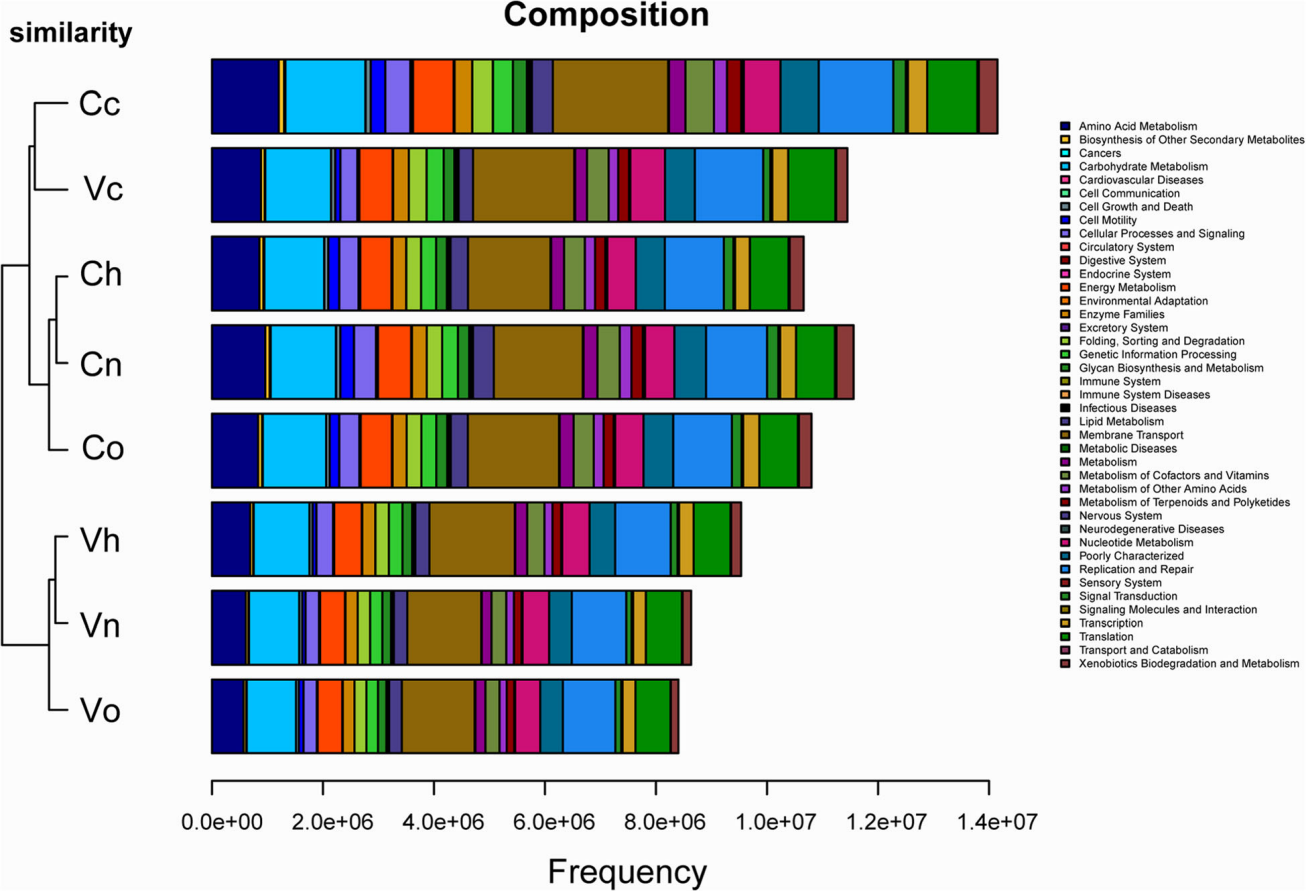
Functional prediction of vaginal and cervical microbiota. The cluster tree and functional bar plot are combined. The sample cluster tree based on Bray-Curtis is shown on the left, in which the length of the branch represents the evolutionary distance between groups. Horizontal bars represent the functional abundance composition of microbiota in each group. Vn, Vo, Vh and Vc represent the vaginal microbiota of groups N (the normal control group), O (the other hrHPV group), H (the HPV16/18 group) and C (the cancer group), respectively. Cn, Co, Ch and Cc represent the cervical microbiota of groups N, O, H and C, respectively

We observed that the microbial functions at cervix were more complicated and active than those in vagina regardless of HPV status. Some special functions involving the amino acid metabolism, carbohydrate metabolism, membrane transport, replication and repair, and gene information processing were relatively more vigorous at cervix (Fig. 7). These functions might be important for virus replication, integration and development of cervical lesions. In addition, function of the cervical and vaginal microbiota in cancer patients were more abundant than those of non-cancer patients. It is necessary to further study the relationship between the function of cervical microbiota and carcinogenesis.

## Discussion

With the rapid development of microbial detection methods, especially the application of NGS, we are increasingly aware of the importance of microorganisms to human health [13, 14]. Certain members of bacteria in the lower genital tract are believed to be beneficial for women against infection and pathogenesis. Healthy vagina is more commonly associated with low microbial diversity and dominated by one or a few species of *Lactobacillus* [3, 15, 16]. The primary defense mechanisms of the lower genital mucosa are antimicrobial peptides, a pH of less than 4.5, and a microbial community dominated by *Lactobacillius*. An imbalance in these defenses could result in pathological alterations of the lower genital environment [7].

HPV is a unique health concern because persistent hrHPV infection may lead to precancer, which, if left untreated, may progress to cervical cancer. In most cases, the immune system clears the virus on its own within 6–18 months [17, 18]. It takes a long time from HPV infection to the development of cancer, which gives us oppurtunity to prevent this deterioration process. Over the past decade, evidence has suggested that the vaginal microbiota also plays a role in cervical carcinogenesis [19]. Emerging studies have revealed associations between the *Lactobacillus-*depleted vaginal microbiota and HPV infection and persistence [20]. Previous studies on the lower genital microbiota only focused on the vaginal microbiota or did not separate the cervical and vaginal flora, simply called it the “cervicovaginal microbiota” [21–23], which may be because it is generally believed there is no difference between the vaginal and cervical microbiota and that contamination is difficult to avoid when separately sampling.

The highlight of our study is that this is the first study that has discussed the distinction between cervical and vaginal microbiota of hrHPV(+) women in China. We were enlightened by the study of Peking University Shenzhen Hospital in 2017 [11], which broke the idea that the upper genital tract was sterile and revealed differences in the microbiota of different parts of the female reproductive tract. They systematically sampled discharges at six sites in the female genital tract from a large cohort of Chinese women of reproductive age. The six parts are the lower third of the vagina, the posterior fornix, the cervical canal, the endometrium, the left/right fallopian tubes and the peritoneal fluid from the Douglas pouch. They clarified that at the phylum level, *Firmicutes* dominated the lower reproductive tract, in contrast to the large proportions of *Proteobacteria*, *Actinobacteria* and *Bacteroidetes* in the upper reproductive tract. We adopted the sampling methods in this study, and cervical discharges were strictly taken from the cervical canal to avoid contamination. Similarly, our research revealed that the cervical microbiota consisted of a lower percentage of *Firmicutes* and a much higher percentage of *Proteobacteria* than the vagina. *Proteobacteria* accounted for approximately 1 % of the normal vaginal microbiota but more than 10 % of the normal cervical microbiota, demonstrating itself to be a particular phylum in the cervix. We speculate a lower percentage of *Firmicutes-Lactobacillius* affects the production of lactic acid and hydrogen peroxide [15]; thus, the pH of the cervix tends to be higher than that of the vagina, which further changes the composition of other strains.

*Proteobacteria* is a special phylum in the cervix rather than the vagina. Are particular genera of *Proteobacteria* associated with hrHPV infection or cervical cancer? There have been no such studies on cervical *Proteobacteria* and hrHPV infection. *Proteobacteria* [24] comprise quite a large community and can be divided into α-, β- and γ-*Proteobacteria*. We noticed that *Pseudomonas* was more prevalent in hrHPV(+) samples, especially the cancerous cervix. *Pseudomonas* belongs to the γ-*Proteobacteria* and has long been believed to be an opportunistic pathogen of the human urogenital system. Jeff [25] studied the role of infectious factors in cervical cancer using *Pseudomonas aeruginosa* as the bacterial tool and *Lactobacillus* as a control. They observed upregulated expression of integrins in cervical cancer tissues and found that *Pseudomonas aeruginosa* could promote the expression of integrins in cervical cancer cell lines, while the control group of *Lactobacillus* showed no change. This result indicates the potential role of *Pseudomonas* in promoting the development of cervical lesions. This is the only basic experimental study on *Pseudomonas* pathogenesis in cervical lesions. Of course, more research is needed to further discuss the mechanism in detail.

Another genus of α-*Proteobacteria*, *Sphingomonas,* was more frequent in the normal cervix and infrequent in the hrHPV(+) and cancerous cervix. Studies on *Sphingomonas* that we have seen are mainly distributed in the field of environmental science. We have not yet seen any studies on *Sphingomonas* in the human reproductive system. *Sphingomonas* are often isolated from petroleum-contaminated soils due to their unique abilities to degrade polycyclic aromatic hydrocarbons (PAHs) [26], which have been recognized as definite carcinogens. Another study [27] suggests that both HPV infection and PAHs are critical factors in the development of cervical cancer. PAHs have the potential to coordinate with HPV to aggravate carcinogenesis at all stages. Therefore, it is not clear whether *Sphingomonas* could play a protective role in the development of cervical lesions by degrading PAHs, which is worthy exploring in further studies. If this hypothesis becomes a reality, we may be able to prevent HPV infection and cervical lesions from another perspective.

Compared to *Proteobacteria*, BV-related anaerobes showed similar changes in the hrHPV(+) vagina and cervix and did not show cervical particularity. *Sneathia* has the most sensitive association with hrHPV infection, regardless of HPV subtypes. *Sneathia* was the first genus to be identified as a target bacteria for HPV infection early in 2013 [9]. Intriguingly, *Sneathia* is the only bacteria that is enriched in the genital tract throughout the process of cervical carcinogenesis regardless of HPV subtypes [28]. By comparison, *Gardnerella*, *Prevotella* and *Atopobium* were more prevalent mainly in HPV16/18(+) or cancerous vagina/cervix. That is, women with HPV16/18(+) have a more disturbed genital microenvironment, which may explain why HPV16/18 are most carcinogenic. By comparing BV-associated anaerobes with certain strains of *Proteobacteria*, such as *Sphingomonas* and *Pseudomonas*, we reconfirmed that community differences existed between the cervical and vaginal microbiota. It is of great significance to deeply analyze the difference between vaginal and cervical microbiota, to find the cervical target genus and to explore their functional mechanism to better prevent HPV infection.

## Conclusion

This is the first study that has paid special attention to the cervical microbiota of hrHPV(+) Chinese women and distinguished it from the vaginal microbiota. The results revealed that *Proteobacteria* was a particular phylum in the cervix than in the vagina. *Sphingomonas,* which belongs to α-*Proteobacteria,* has the potential to play a protective role in hrHPV infection, while *Pseudomonas* in γ-*Proteobacteria* is positively associated with hrHPV infection and cervical cancer. These findings will provide new ideas for the prevention of hrHPV from the perspective of microecology. This project also has some shortcomings, such as the limitations of cross-sectional studies and small sample sizes. Large-scale prospective clinical trials need to be implemented in the future to discover the changes in microbiota longitudinally in the chronic process of persistent hrHPV infection and to explore the predictive and therapeutic value of specific genera on hrHPV infection and cervical lesions. This is a microbiological age, and microecological prevention and therapy will become possible.

## Methods

### Study cohort and sample collection

The study was conducted in accordance with the Declaration of Helsinki and its current amendments, and the protocol was approved by the medical ethics committee of Peking University First Hospital. All subjects provided written informed consent, and there was no financial compensation. One hundred women of reproductive age in the gynecological clinic were recruited and divided into four groups according to routine cervical cancer screening results. The normal group (Group N) comprised 20 women whose Thinprep cytologic test (TCT) and HPV were both negative. The HPV16/18 group (Group H) comprised 38 hrHPV16/18 (+) women, and colposcope biopsies showed no cancerous lesions. The other hrHPV group (Group O) comprised 32 women with hrHPV except for HPV 16/18, and biopsies showed no cancerous lesions. The cancer group (Group C) comprised 10 women with cervical carcinoma.

Inclusion criteria: women of reproductive age; having sexual experience; having regular menstruation; mid-follicular phase; no usage of any medications within 1 week; no vaginal douching, cervical treatment or sexual intercourse within 72 h [29]. Exclusion criteria: women during pregnancy, lactation or menopause and women with chronic diseases who need long-term medication.

All participants were required to complete questionnaires, including age, educational level, occupation, economic condition, hygiene practices, sexual activity, history of vaginitis and cervical cancer screening results. Discharges of the vagina and cervix were collected and reserved with separate sterile cotton swabs and Eppendorf (EP) tubes containing normal saline, stored at − 80 °C and transported on dry ice to Sangon Biotech-Shanghai for NGS. Vaginal discharge was obtained from up to one-third of the vagina. To avoid contamination, the surface discharge of the cervix was wiped before formal sampling, and a sterile cotton swab was directly and strictly inserted into the cervical canal to acquire the discharge of the cervix [11]. Another swab was used for smearing, Gram staining and oil lens observation to evaluate the vaginal microecology and numbers of cervical leukocytes. The Nugent score was adopted to diagnose BV (Nugent score 7–10: BV; 4–6: BV intermediate; 1–3: normal). Vulvovaginal candidiasis (VVC) was indicated when hyphae or spores were discovered, and Trichomonas vaginitis (TV) was indicated when Trichomonas was seen under an oil lens. Cervicitis was indicated when the average number of leukocytes was more than 10/high power field. TCT was interpreted by two experienced cytologists. HPV DNA was extracted from exfoliated cells, amplified by PCR, and hybridized on a low-density gene chip with a fixed nucleic acid probe (Kaipu Biotechnology, Guangdong, China), which we used to determine the types of HPVs.

### DNA extraction and 16S rRNA V3-V4 gene sequencing

Microbiota sequencing was performed targeting the V3-V4 region of the 16S rRNA genes using the Illumina MiSeq platform. DNA was extracted according to the instructions of the OMEGA E.Z.N. ATM Mag-Bind Soil DNA Kit. DNA integrity was detected by agarose gel electrophoresis. The V3-V4 region of the 16S rRNA genes was amplified by polymerase chain reaction (PCR) with a universal forward primer and unique barcode primer [30] (V3-341F: CCCTACACGACGCTCTTCCGATCTG (barcode) CCTACGGGNGGCWGCAG; V4-805R: GACTGGAGTTCCTTGGCACCCGAGAATTCCA (barcode) GACTACHVGGGTATCTAATCC).

The first amplification was performed under the following conditions: 3 min of denaturation at 94 °C; 5 cycles of denaturation at 94 °C for 30 s, annealing at 45 °C for 20 s, and elongation at 65 °C for 30 s; 20 cycles of denaturation at 94 °C for 20 s, annealing at 55 °C for 20 s, and elongation at 72 °C for 30 s; and a final extension at 72 °C for 10 min. Illumina bridge PCR compatible primers were introduced in the second amplification as follows: 3 min of denaturation at 95 °C; 5 cycles of denaturation at 94 °C for 20 s, annealing at 55 °C for 20 s, and elongation at 72 °C for 30 s; and a final extension at 72 °C for 10 min. Amplicons were purified using AMPure XP beads, and DNA quantitation was performed using a Qubit 3.0 DNA Kit, 10 ng of DNA extracted from each sample was sequenced using the Illumina MiSeq 2 × 300 bp platform.

Raw data were processed as follows: remove joint sequences of primers, splicing sequences according to the overlap, identify sample data by barcode and remove chimeras and nonspecific sequences to achieve quality control. Operational taxonomic unit (OTU) clustering was performed at a 97% similarity level. Software used: Cutadapt, PEAR [31], Prinseq [32], Usearch [33] and Uchime [34].

### Bioinformatics analysis

For convenience of understanding, the vaginal microbiota of the four groups (Group N O H C) was abbreviated Vn, Vo, Vh, and Vc, and the cervical microbiota of the four groups was abbreviated Cn, Co, Ch, and Cc.

Microorganism taxonomy: Species taxonomy was performed based on the Ribosomal Database Project (RDP) classifier [35] and Bergey’s taxonomy. Microbes were analyzed at six levels, namely, domain, phylum, class, order, family and genus. Taxonomy diagrams were drawn using R software [36].

Alpha and beta diversity: Alpha diversity was used to evaluate the diversity of species within each sample or each group. Calculated shannon index of each group. The greater the Shannon value was, the higher the diversity of the community. Beta diversity was used to measure the evolutionary distances between different samples or groups evaluated by UniFrac distance. The distance of UniFrac is between 0 and 1, and the higher the value is, the further the evolutionary distance. Software used: Muscle [37], FastTree [38], mothur [39] and R [36].

Linear discriminant analysis effect size (LEfSe) analysis: LEfSe analysis could best explain the community difference in each group. Statistical methods: Kruskal-Wallis rank-sum test, (unpaired) Wilcoxon rank-sum test and linear discriminant analysis. Software used: LEfSe [40].

Functional analysis: Sequences acquired by NGS were translated to corresponding functions using R software by paralleling the Kyoto Encyclopedia of Genes and Genomes (KEGG) databases [41]. Software used: PICRUSt [42].

### Statistical analysis of demographics data

SPSS 19.0 and GraphPad Prism 5 were used for data analysis. Age was analyzed by ANOVA, and the other items were analyzed by the chi-square test or Fisher’s exact probability test. For the analysis of genus differences, the Mann-Whitney test and Wilcoxon signed rank test were used for the comparative analysis within and between the vaginal and cervical microbiota, respectively. *P* < 0.05 was statistically significant.

## Data Availability

The datasets used and/or analyzed during the current study are available from the corresponding author on reasonable request.

## Abbreviations

HPV: Human papilloma virus
hrHPV: high-risk HPV
NGS: Next-generation sequencing technology
SIL: Squamous intraepithelial lesion
TCT: Thin-prep cytology test
Ascus: Atypical squamous cell of undetermined significance
LSIL: low-grade SIL
HSIL: high-grade SIL
ASC-H: Atypical squamous cell-cannot exclude HSIL
FIGO: Federation of International Gynecology and Obstetrics
BV: Bacterial vaginosis
KEGG: Kyoto Encyclopedia of Genes and Genomes
PAH: Polycyclic aromatic hydrocarbon
EP: Eppendorf
VVC: Vulvovaginal candidiasis
TV: Trichomonas vaginitis
PCR: Polymerase chain reaction
OTU: Operational Taxonomic Unit
RDP: Ribosomal Database Project
LEfSe: Linear discriminant analysis Effect
